# Case Report: Novel Biallelic Mutations in *ARMC4* Cause Primary Ciliary Dyskinesia and Male Infertility in a Chinese Family

**DOI:** 10.3389/fgene.2021.715339

**Published:** 2021-07-30

**Authors:** Yang Gao, Chuan Xu, Qing Tan, Qunshan Shen, Huan Wu, Mingrong Lv, Kuokuo Li, Dongdong Tang, Bing Song, Yuping Xu, Ping Zhou, Zhaolian Wei, Fangbiao Tao, Yunxia Cao, Xiaojin He

**Affiliations:** ^1^Department of Obstetrics and Gynecology, Reproductive Medicine Center, The First Affiliated Hospital of Anhui Medical University, Hefei, China; ^2^NHC Key Laboratory of Study on Abnormal Gametes and Reproductive Tract, Anhui Medical University, Hefei, China; ^3^Key Laboratory of Population Health Across Life Cycle, Anhui Medical University, Ministry of Education of the People's Republic of China, Hefei, China; ^4^Anhui Provincial Human Sperm Bank, The First Affiliated Hospital of Anhui Medical University, Hefei, China; ^5^Anhui Province Key Laboratory of Reproductive Health and Genetics, Anhui Medical University, Hefei, China

**Keywords:** male infertility, primary ciliary dyskinesia (Kartagener syndrome), ARMC4, oligoasthenoterazoospermia, ICSI

## Abstract

Primary ciliary dyskinesia (PCD) is a clinically and genetically heterogeneous ciliopathy affecting the cilia and sperm flagella. Mutations in genes related to the structural and functional defects of respiratory ciliary axoneme have been reported to be the predominant cause of this symptom; however, evidence regarding male infertility and genotype–phenotype associations between some of these genes and flagellar axoneme remains unclear. Here, we reported a male patient from a non-consanguineous Chinese family who exhibited left/right body asymmetry and oligoasthenoterazoospermia factor infertility. Novel compound heterozygous mutations in *ARMC4* (NM:018076: c.2095C>T: p. Gln699^*^; c.1679C>T: p. Ala560Val) were identified in this patient, and his parents were a heterozygous carrier for the mutations. Morphological and ultrastructural analysis of the spermatozoa from the man showed aberrant sperm flagella with axonemal disorganization and outer dynein arm (ODA) loss. In addition, immunofluorescence analysis of the spermatozoa from the proband and a control man revealed a significant lower expression of ARMC4 protein due to pathogenic mutations. Therefore, our findings help to expand the spectrum of *ARMC4* pathogenic mutations and linked biallelic *ARMC4* mutations to male infertility for the first time.

## Introduction

Primary ciliary dyskinesia (PCD, MIM 244400) is a genetically heterogeneous syndrome (Fliegauf et al., 2007), clinically characterized by the presence of chronic airway symptoms, obstructive lung disease, defects in laterality, and infertility (Goutaki et al., 2016). In human, a wide spectrum of recessive inherited genes, predominantly encoding ciliary/flagellar components, has linked PCD to various axonemal ultrastructural abnormalities. To date, more than 40 genes have been reported to cause this syndrome (Lucas et al., 2020). However, most genetic research studies of PCD are based on a cohort of patients with typical symptoms diagnosed at the early stage of life, which consequently ignore the genetic relationship between male infertility and PCD (Sironen et al., 2019; Guo et al., 2020).

In this study, we identified novel biallelic *ARMC4* mutations in a Chinese male patient with PCD and infertility associated with oligoasthenoterazoospermia. The *ARMC4* gene encodes a component of the outer dynein arm-docking complex (ODA-DC) that mediates ODA targeting and/or anchoring onto the doublet microtubule. Mutations in *ARMC4* gene have been reported to cause PCD in human, mice, and zebrafish (Hjeij et al., 2013). Moreover, Hjeij et al. and Onoufriadis et al. both demonstrated that *ARMC4*-related PCD showed defects of the left–right axis and distal ODAs of the respiratory cilia (Hjeij et al., 2013; Onoufriadis et al., 2014). However, no sperm-related examinations were performed in these cases, and whether *ARMC4* mutations have effects on sperm flagella structure remains unclear. This is the first report of *ARMC4* mutations that associates with oligoasthenoterazoospermia in human and expands the spectrum of known *ARMC4* mutations.

## Patients and Methods

The proband was a 30-year-old man from a non-consanguineous Han Chinese family. He was recruited to identify the genetic risk factors at the Reproductive Center of the First Affiliated Hospital of Anhui Medical University due to his 3–4 years of primary infertility combined with dexiocardia symptom in 2020. The routine clinical examinations excluded the usual causes for male infertility including developmental effects, hormone levels, chromosomal aberration, and Y chromosome microdeletion. However, semen analyses showed oligoasthenospermia, which is clinically considered as a risk factor for infertility. Moreover, chest X-ray revealed left/right body asymmetry of the proband, but the parents are normal. Based on these findings, whole-exome sequencing (WES) was performed on the proband to identify genetic causes as previously described (He et al., 2020). Sanger sequencing was further used to validated pathogenic variants in his family (Supplementary Table 2). Immunofluorescence and transmission electron microscopy (TEM) analysis were performed to detect changes of the spermatozoa of the proband. All subjects were enrolled in accordance with the protocol approved by the Ethics Review Committee of the First Affiliated Hospital of Anhui Medical University and signed the informed consent.

## Results

### Identification of Novel Biallelic Mutations in ARMC4

Whole-exome sequencing was performed on the man, and compound heterozygous variants affecting exonic regions or splice sites were screened based on a recessive disease model. First, according to our filtering criteria, variants present in the 1000G, ExAC, and gnomAD databases with allele frequency >0.01 were removed. Second, we screened the variants predicted to be deleterious by more than three of the four biological systems (SIFT, PolyPhen-2, MutationTaster, CADD). The variant with CADD score higher than 20 was defined as deleterious. Third, genes associated with the ciliary phenotype were retained for further verification.

**Figure 1:**
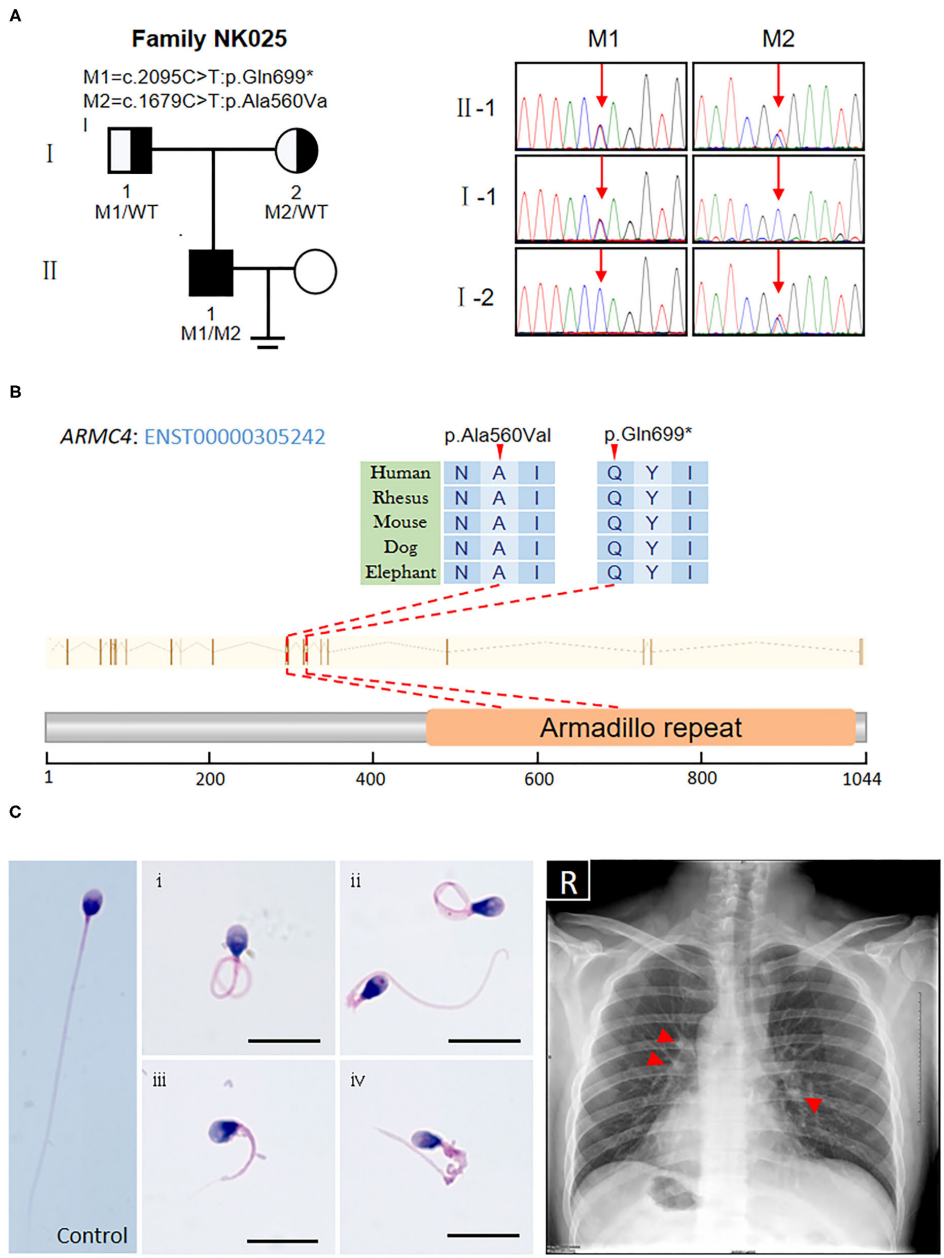
Identification of novel biallelic ARMC4 mutations in a Chinese man with left/right body asymmetry and oligoasthenospermia-associated male infertility. **(A)** The pedigree of the investigated men affected by biallelic mutations in *ARMC4*. Sanger sequencing results are shown on the right. Red arrows indicated the mutated positions. **(B)** Schematic of the positions of mutations identified in the *ARMC4* genome and protein sequence. One of the mutations introduces a stop codon which leads to a premature stop and the other is a missense mutation exchanging alanine with valine at position 560. Both mutated residues are located in the evolutionarily conserved region. **(C)** Morphology of the spermatozoa from *ARMC4*-mutated men shows the MMAF phenotype, defined by coiled (i), bent (ii), short (iii), and irregular-caliber flagella (iv). Scale bar = 10μm. Chest X-ray of *ARMC4*-mutated men shows randomization of situs inversus (dextrocardia, gastric bubble right, liver on the left) and paracardial opacities with cystic lesions, suggesting atelectasis of the middle lobe and bronchiectasis (red arrows).

Based on the criteria, we found novel putative compound heterozygous variants in the *ARMC4* gene (Table 1). Sanger sequencing confirmed the presence of the variants in the man, whereas his parents were heterozygous carriers (Figure 1A). Both of the variants were located in a conserved ARM domain of the ARMC4 protein, and the affected residues were highly conserved among different species (Figure 1B).

**Table 1:** Overview of the identified *ARMC4* mutations.

	**NK025 II-1**	
Positions	Chr10:28228828	Chr10:28233215
cDNA mutation	c.C2095T	c.C1679T
Protein alteration	p. Q699X	p. A560V
Mutation type	Stopgain	Missense variant
**Allele frequency**
1000G	NA	NA
ExAC03	NA	0.000025
GnomAD exome	NA	0.00003981
**Function prediction**
SIFT	NA	D
PolyPhen-2	NA	D
MutationTaster	D	D
CADD	52	24.1

*RefSeq accession number of ARMC4 is NM_018076. NA, not available*.

### Clinical Description and Good Intracytoplasmic Sperm Injection Outcome of the *ARMC4*-Mutated Patient

Routine semen analysis of NK025 II-1 showed oligoasthenospermia (sperm concentration: 7.5 × 10^6^/ml, progressive rate: 20%) (Supplementary Table 1) (Cooper et al., 2010). Morphological analysis of the spermatozoa from NK025 II-1 revealed multiple morphological abnormalities of the sperm flagella (MMAF) phenotype (Figure 1C), of which only 20.5% (41/200) of the spermatozoa exhibited normal tail morphologies, while coiled flagella, short flagella, angled flagella, and absent flagella of the spermatozoa were frequently observed (38, 20, 11.5, and 8%, respectively) (Supplementary Table 1) (Auger et al., 2016).

Next, we performed WES analysis on the spouse of the patient to exclude pathogenic *ARMC4* variants before choosing intracytoplasmic sperm injection (ICSI) treatment. During the ICSI process, she received ovarian stimulation by the GnRH antagonist protocol. The rFSH was administered at a dose of 187.5 IU on day 2 of the menstrual cycle and 187.5 IU per day for 10 days. Subsequently, 18 meiosis II oocytes were injected with spermatozoa from NK025 II-1 and 15 two-pronuclear zygotes were observed on day 1. All the zygotes underwent normal cleavage. We finally acquired nine good quality embryos and frozen these on day 5 and day 6. One viable embryo was transferred in a frozen-thawed embryo cycle, and clinical pregnancy was diagnosed by transvaginal ultrasonography 30 days after transplantation fortunately (Table 2).

**Table 2:** ICSI outcomes of the *ARMC4-*mutated patient.

	**II-1**
Male age (years)	30
Female age (years)	30
No. of ICSI cycles	1
No. of oocytes injected	18
Fertilization rate (%)	83.3 (15/18)
Cleavage rate (%)	100 (15/15)
Eight-cell embryo development rate (%)	66.7 (10/15)
Blastocyst formation rate (%)	60 (9/15)
No. of frozen-thawed embryo transfer cycles	1
Number of embryos transferred	1
Implantation rate (%)	100
Clinical pregnancy	Y
Live birth	Y

*ICSI, intracytoplasmic sperm injection; Y, yes*.

### The MMAF Phenotype Was Linked to Lower ARMC4 Protein Expression and Ultrastructural Defects in the Spermatozoa From ARMC4-Mutated Men

**Figure 2:**
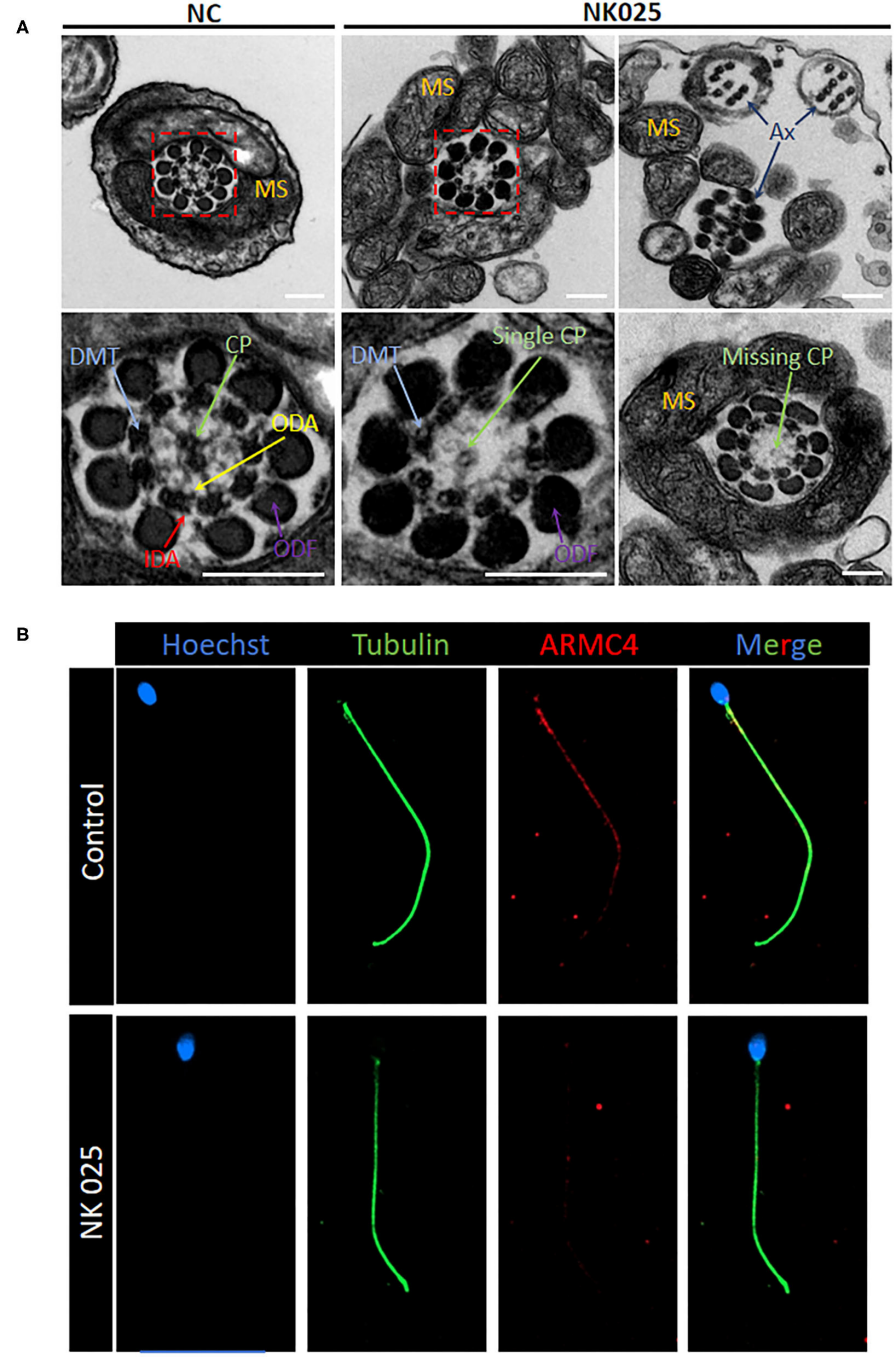
The *ARMC4*-mutated man exhibits lower ARMC4 protein expression and ultrastructural defects in axonemal structure. **(A)** Cross-sections of sperm flagella from the control man and the *ARMC4*-mutated men. Compared with those in normal sperm flagella, the axoneme of *ARMC4*-mutated men exhibited multiple defects of the axoneme, including absent of outer/inner dynein arms, CP defects (“9+0” “9+1”), and disorder of axoneme. Scale bars = 200 nm. **(B)** ARMC4 staining was present mainly in the midpiece and principal piece of spermatozoa flagella from the control man, but absent or had a significant decrease in the spermatozoa from the *ARMC4*-mutated men. CP, central microtubule pair (green arrows); ODA, outer dynein arm (yellow arrows); IDA, inner dynein arm (red arrows); MS, mitochondrial sheath; DMT, (blue arrows); ODF, outer dense fiber (purple arrows).

The electron microscopy was used to evaluate the ultrastructural changes of the spermatozoa from NK025 II-1. High-resolution morphological analysis using scanning electron microscopy (SEM) confirmed that the spermatozoa of the patient displayed multiple sperm flagella abnormalities mainly with coiled and short flagella (≥58%) (Supplementary Figure 1). Moreover, TEM found aberrant axonemal cross-sectional structures of the spermatozoa of the patient (loss of the ODAs and defects of the central pair microtubules) compared with the spermatozoa from normal control (Figure 2A). To better understand the pathogenicity of *ARMC4*-mutated residues at the molecular level, we performed immunofluorescence for spermatozoa from NK025 II-1 and unaffected individuals. In normal spermatozoa, immunostaining of the ARMC4 protein was located along the length of the sperm flagella. However, ARMC4 immunostaining is markedly reduced along the full length of the flagella in the spermatozoa from NK025 II-1, which was consistent with the ODA defects observed (Figure 2B).

## Discussion

Dysfunction of the cilia will lead to recurrent respiratory tract infections from early infancy, chronic rhinosinusitis, bronchiectasis, and otitis media (Mirra et al., 2017). Moreover, half of the patients had situs inversus, which is thought to be due to nodal cilia defects during embryonic development. Although advances in genetic etiology have identified pathogenic mutations in more than 40 flagella and motile cilia-associated genes, little is known about the fertility of such patients at their reproductive age not to mention the detailed sperm phenotypes of male patients (Goutaki et al., 2016).

In this study, we identified novel biallelic mutations in the PCD-related gene *ARMC4* that might be responsible for human oligoasthenoterazoospermia factor infertility. As early as 2013, two main research studies have described the genotype–phenotype association between PCD and *ARMC4* gene in a species conserved model. Hjeij et al. found that the absence of ARMC4 localization would lead to a significant reduction of ODAs in the respiratory cilia axonemes from patients with truncating mutations (Hjeij et al., 2013). Besides, Onoufriadis et al. found that ARMC4 transcript levels were upregulated significantly after ciliogenesis but undetectable in non-ciliated human bronchial epithelial cells (Onoufriadis et al., 2014). These findings suggested that the ARMC4 protein is required for a late step in proper targeting and anchoring of ODAs.

Motile cilia and sperm flagella are evolutionarily conserved and related organelles across species. In humans, respiratory motile cilia and sperm flagella share a common axoneme structure, consisting of a central microtubule pair surrounded by nine peripheral microtubule doublets (Lehti and Sironen, 2017). In addition, a variety of microtubular protein complexes, including radial spokes, nexin–dynein regulatory complexes, and inner and ODAs, are attached along the entire length of the axoneme (Toure et al., 2020). Although a role for *ARMC4* in sperm flagella function has not been reported, the RNA expression profile has been inferred to be involved in sperm cells (Sironen et al., 2019).

The C-terminal of ARMC4 protein contains the well-known armadillo repeat motifs (ARMs). It is reported that tandem ARM-repeat sequence fold together as a superhelix, forming a conserved three-dimensional structure to interact with binding partners (Coates, 2003). Notably, the ARMs containing proteins are known to be involved in various processes, including signal transduction and cytoskeletal regulation (Coates, 2003; Cheng et al., 2013). In this present study, the identified novel biallelic *ARMC4* mutations were both located in this region of the protein, consistent with the mutations found in previous studies (Hjeij et al., 2013; Onoufriadis et al., 2014). It is therefore highly suggested that the mutations may influence the protein-binding capabilities of ARMC4.

To verify the impact of mutations in the spermatozoa from this ARMC4-mutated man, we performed immunofluorescence analysis by using rabbit polyclonal anti-ARMC4 antibodies (HPA037829, Atlas Antibodies, Stockholm, Sweden). Immunofluorescence microscopy revealed that the ARMC4 protein localized mainly to the midpiece and principal piece of the sperm flagella in control sperm cells, and *ARMC4*-mutated sperm cells showed obviously a lower expression. We also performed cross-sectional analysis of the sperm flagella in an unaffected individual and *ARMC4*-mutated male patient by TEM. We found ODA abnormalities in most of the analyzed cross-sections of the patient, indicating that ARMC4 mutations affect ODA attachment along the entire axonemal length. In addition, previous studies have reported that men with PCD who were unable to reproduce naturally may have poorer outcomes with ICSI treatment (Yildirim et al., 2009; McLachlan et al., 2012). However, the specific mutations of these PCD patients were unknown. Here, we reported successful ICSI outcome and consequent live birth was achieved within first transplantation with sperm from an *ARMC4*-mutated PCD patient. We believe that it is of great importance to investigate the specific gene mutations on PCD patients and their fertility status or treatment outcomes to improve counseling and treatment of infertility in PCD patients.

Besides, we noticed that semen analysis of the *ARMC4*-mutated man also showed oligospermia (sperm count <15 million per ml); however, no experimental information was obtained to explain this phenomenon. Based on the observation that the ARMC4 protein is clearly expressed in the epididymal epithelium (The Human Protein Atlas: http://www.proteinatlas.org/), we speculated here that *ARMC4*-mutated men may have deficiency in epididymal cilia function (Ichioka et al., 2006). However, the proper mechanism of each gene and the effects of their abnormalities on male infertility are yet to be explored.

In conclusion, we identified novel pathogenic mutations in *ARMC4* leading to low expression of ARMC4 protein in sperm flagella, which might cause male infertility with oligoasthenoterazoospermia and impact the ODA ultrastructure of mutated spermatozoa. Moreover, our clinical and genetic findings help to expand the spectrum of the *ARMC4* gene and provide a deeper insight to our knowledge of the pathogenic effect of *ARMC4* mutations in the reproductive system.

## Data Availability Statement

The original contributions presented in the study are included in the article/Supplementary Materials, further inquiries can be directed to the corresponding author/s.

## Ethics Statement

The studies involving human participants were reviewed and approved by the Ethics Review Committee of the First Affiliated Hospital of Anhui Medical University. The patients/participants provided their written informed consent to participate in this study. Written informed consent was obtained from the individual(s) for the publication of any potentially identifiable images or data included in this article.

## Author Contributions

XH and YC designed the overall study concept. YG, XH, and YC wrote the manuscript. YG, CX, and QT performed the experiments. ML, KL, QS, HW, and DT analyzed the genetic data. BS, YX, FT, PZ, and ZW provided clinical data and supplied biological materials. All authors edited and reviewed the manuscript.

## Conflict of Interest

The authors declare that the research was conducted in the absence of any commercial or financial relationships that could be construed as a potential conflict of interest.

## Publisher's Note

All claims expressed in this article are solely those of the authors and do not necessarily represent those of their affiliated organizations, or those of the publisher, the editors and the reviewers. Any product that may be evaluated in this article, or claim that may be made by its manufacturer, is not guaranteed or endorsed by the publisher.
