# Supplementary material for: Case Report: Novel Biallelic Mutations in ARMC4 Cause Primary Ciliary Dyskinesia and Male Infertility in a Chinese Family
Source: Front Genet. 2021 Jul 30;12:715339. doi: 10.3389/fgene.2021.715339 (PMC8362595; doi:10.3389/fgene.2021.715339)
Supplement: Supplementary file 1 [file Data_Sheet_1.docx]

**NK025**

**NC**

**
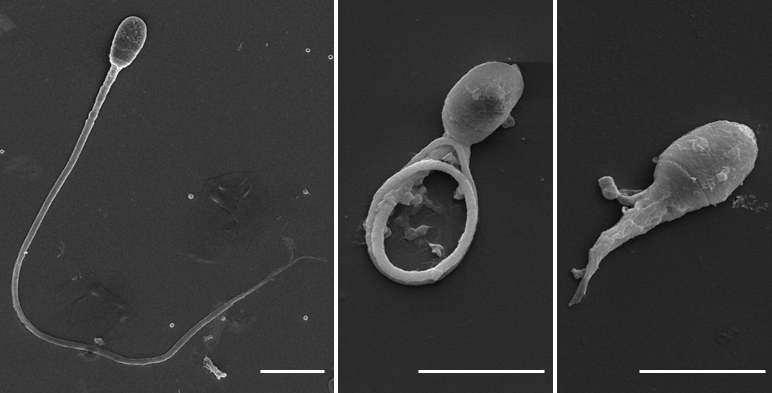
Supplementary figure 1: Morphology of spermatozoa from control man and patient under SEM.** The scanning electron microscopy analysis showed that the spermatozoa of patient NK025 were mainly coiled and short flagella. Scalebar:10 μm.

**Supplementary table 1. Sperm analyses of the *ARMC4*-mutated patient.**

| **Semen Parameters** | **Family1 II-1** | **Reference Values** |
| --- | --- | --- |
| Semen volume (ml) | 1.6 | >1.5 |
| Sperm concentration (10^6^/mL) | 7.5 | >15.0 |
| Total sperm count (10^6^) | 12 | >39.0 |
| Motility (%) | 34 | >40.0 |
| Progressive motility (%) | 20 | >32.0 |
| **Sperm Morphology** |  | |
| Absent flagella (%) | 8 | <5.0 |
| Short flagella (%) | **22** | <1.0 |
| Coiled flagella (%) | **38** | <17.0 |
| Angulation flagella (%) | 11.5 | <13.0 |
| Normal flagella (%) | 20.5 | >23.0 |
| Lower and upper reference limits according to the World Health Organization (WHO) standards and distribution range of morphologically abnormal spermatozoa observed in fertile individuals^13^ and the distribution range of morphologically abnormal spermatozoa observed in fertile individuals^14^. | | |

**Supplementary table 2. Primers used for verification of *ARMC4* mutations.**

| **Primer Names** | **Primer Sequences (5'-3')** | **Tm** |
| --- | --- | --- |
| M1-F | TGTTAAGCTTGGACTATGGTGA | 57.5 |
| M1-R | ACTTGAAGCCAGGGGTTTG | 60 |
| M2-F | TTCTTAAGGGCAAGCGATACAG | 56.54 |
| M2-R | GGTTACATATTCGCCACTCATC | 56.54 |
